# Induction of Macrophage Function in Human THP-1 Cells Is Associated with Rewiring of MAPK Signaling and Activation of MAP3K7 (TAK1) Protein Kinase

**DOI:** 10.3389/fcell.2016.00021

**Published:** 2016-03-30

**Authors:** Erik Richter, Katharina Ventz, Manuela Harms, Jörg Mostertz, Falko Hochgräfe

**Affiliations:** Junior Research Group Pathoproteomics, Competence Center Functional Genomics, University of GreifswaldGreifswald, Germany

**Keywords:** chemical proteomics, kinomics, phosphoproteome, monocyte-to-macrophage differentiation, kinome, signaling, THP-1 cells

## Abstract

Macrophages represent the primary human host response to pathogen infection and link the immediate defense to the adaptive immune system. Mature tissue macrophages convert from circulating monocyte precursor cells by terminal differentiation in a process that is not fully understood. Here, we analyzed the protein kinases of the human monocytic cell line THP-1 before and after induction of macrophage differentiation by using kinomics and phosphoproteomics. When comparing the macrophage-like state with the monocytic precursor, 50% of the kinome was altered in expression and even 71% of covered kinase phosphorylation sites were affected. Kinome rearrangements are for example characterized by a shift of overrepresented cyclin-dependent kinases associated with cell cycle control in monocytes to calmodulin-dependent kinases and kinases involved in proinflammatory signaling. Eventually, we show that monocyte-to-macrophage differentiation is associated with major rewiring of mitogen-activated protein kinase signaling networks and demonstrate that protein kinase MAP3K7 (TAK1) acts as the key signaling hub in bacterial killing, chemokine production and differentiation. Our study proves the fundamental role of protein kinases and cellular signaling as major drivers of macrophage differentiation and function. The finding that MAP3K7 is central to macrophage function suggests MAP3K7 and its networking partners as promising targets in host-directed therapy for macrophage-associated disease.

## Introduction

Cellular differentiation is a fundamental process in development that is triggered by internal or external stimuli resulting in cells with increased degree of specialization with regard to its respective progenitors. On the molecular level cell transformation is founded on signaling networks that transduce stimuli from the cell surface to modulators by protein phosphorylation and dephosphorylation as the most important posttranslational modification in transmission and integration of signals in cellular networks.

The mononuclear phagocyte system (MPS) represents an impressive example of how cell differentiation generates functional and phenotypic diversity. The common origin of cells of the MPS are hematopoietic stem cells located in the bone marrow that transform through different myeloid progenitor intermediates to monocytes that enter circulation, migrate into tissue and terminally differentiate to replenish resident dendritic cells and macrophages (Geissmann et al., 2010a). Macrophages differ from their monocytic precursors morphologically and are characterized by elevated lysosomal and mitochondrial content as well as in their responsiveness to pathogen-associated molecular patterns—small molecular motifs conserved within a class of microbes (Cohn, 1968; Ross and Auger, 2002). Furthermore, monocytes and macrophages are both critical effectors and regulators of inflammation and the innate immune response e.g., by producing growth factors and cytokines (Geissmann et al., 2010b). The differential degree of functional specialization and adaptive capabilities are attributable to molecular structures that are significantly co-determined by cell signaling components. We hypothesize that major differences exist between the kinomes of monocytes and macrophages and that this has a determining influence in shaping the functional cellular characteristics. Comparative profiling of the kinomes of both cell types could thus increase our understanding in the signal transduction capabilities and assist identification of kinases that are associated with cell type-specific functions.

Analysis of monocyte function and differentiation is still a challenging task. Monocytes permanently sense their environment and react with rapid alteration in phenotype and behavior already during isolation and processing (Auffray et al., 2009). Moreover, limited quantities and heterogeneity of the monocyte population in terms of differentiation stages hinder their analysis. To overcome these drawbacks, a number of human leukemia model cell lines with monocytic characteristics have been established that represent a relative homogenous population and can easily be expanded *in vitro*. These cell lines are blocked at different stages of differentiation but can be released for further progression using specific stimuli (Auwerx, 1991). The human cell line THP-1 is one of the most frequently used model system with monocytic properties (Tsuchiya et al., 1980). THP-1 cells can be differentiated into macrophage-like cells that resemble properties of mature macrophages by activation of protein kinase C (PKC) with phorbol-12-myristate-13-acetate (PMA), ultimately resulting in cells with increased adherence and loss of proliferative activity (Schwende et al., 1996).

Several recent studies employed proteomic techniques that revealed changes in the proteome signature that are associated with differentiation of monocytes to macrophages. Accordingly, increased expression of proteins involved in the suppression of NF-κB including superoxide dismutase 2 (SOD2), the sodium pump subunit alpha-1 (ATP1A1) and a serine peptidase inhibitor (SERPINB2) was highlighted during differentiation of primary human monocytes (Kraft-Terry and Gendelman, 2011). In addition, PMA-induced differentiation of U937 cells is associated with elevated expression of proteins involved in carbohydrate metabolism, antioxidant defense and actin filament rearrangement (Sintiprungrat et al., 2010). Although, these studies shed light on the functional rearrangement on the proteome level, cellular signaling nodes including kinases and phosphatases as the driving forces behind cellular differentiation are underrepresented and hence their impact on monocyte and macrophage properties is not well understood.

Here, we applied a quantitative chemical proteomics strategy for the systematic analysis of the kinome following PMA-triggered differentiation of THP-1 monocytes. We reveal major kinome rearrangement following monocyte differentiation and demonstrate that the macrophage-associated kinase MAP3K7/TAK1 is a central hub in the signal transduction involved in bacterial killing, chemokine production and the differentiation process itself.

## Materials and methods

### Cell culture, SILAC, and differentiation

THP-1 monocytes were purchased from the German collection of microorganisms and cell cultures (DSMZ). Stable isotope labeling with amino acids in cell culture (SILAC, Ong et al., 2002, 2003) was achieved by propagating cells in RPMI 1640 free of L-lysine, L-arginine and L-glutamine (PAA Laboratories) supplemented with 220 μM L-lysine and 144 μM L-arginine in either their light (Lys-0 and Arg-0) or heavy isotope-labeled forms (^13^${\text{C}}_{6}^{15}$N_2_-L-lysine/Lys-8 and ^13^${\text{C}}_{6}^{15}$N_4_-L-arginine/Arg-10) (Silantes), 2 mM L-glutamine (Sigma-Aldrich), 10% heat-inactivated dialyzed fetal calf serum (FCS) (Sigma-Aldrich) and the antibiotics penicillin and streptomycin (Biochrom) at concentrations of 100 units/ml and 100 μg/ml respectively. Cell culturing was performed at 37°C and 5% CO_2_ in a humidified atmosphere. Cells were grown for at least six cell doublings to ensure complete incorporation of labeled amino acids. For differentiation, phorbol-12-myristate 13-acetate (PMA) (Sigma-Aldrich) was added to a final concentration of 100 nM. After 3 days, the PMA supplemented media was removed, cells were washed with PBS and rested in fresh PMA-free media for further 24 h in order to obtain phenotypic characteristics of macrophages (Daigneault et al., 2010).

### Immobilization of kinase inhibitors

Affinity beads were prepared with modifications as described elsewhere (Bantscheff et al., 2007; Wissing et al., 2007). Carbodiimid chemistry was used in order to immobilize the kinases inhibitors SU6668 (Tocris), Purvalanol B (Tocris), and VI16832 (Evotec) to either ECH-Sepharose or EAH-Sepharose (GE) beads. Inhibitors were dissolved in coupling buffer (50/50 sodium phosphate pH 7.0/dimethylformamide) and beads were prepared according to manufacturer's recommendations. Crosslinking was performed in coupling buffer for 24 h at 4°C using N-(3-Dimethylaminopropyl)-N-ethylcarbodiimide hydrochloride (EDC, Sigma-Aldrich) as crosslinker. Beads were extensively washed with coupling buffer and blocked with ethanolamine for 24 h.

### Preparation of cell lysates and enrichment of protein kinases by small molecule affinity chromatography (SMAC)

Undifferentiated THP-1 cells were harvested by centrifugation for 5 min and 130 × g at 4°C and washed twice with phosphate-buffered saline (PBS). Differentiated and undifferentiated THP-1 cells were lysed in buffer containing 50 mM HEPES pH 7.5, 2 M NaCl, 1 mM EDTA, 1 mM EGTA, 0.5% (v/v) TritonX-100, 1 mM PMSF, 50 ng/ml calyculin A, 10 μg/ml leupeptin, 10 μg/ml aprotinin, 10 mM NaF, and 2.5 mM Na_3_VO_4_. Cell lysates were prepared by freeze thawing, followed by sonication. Cell debris was removed by centrifugation for 30 min at 8700 × g at 4°C followed by passing through a 0.45 μm cellulose acetate filter (VWR). Protein concentrations of clarified lysates were determined by Bradford Assay (BioRad).

Heavy and light SILAC lysates with a total protein content of 50 mg were mixed and applied to affinity columns casted from 500 μl of a 50% bead suspension carrying Purvalanol B and SU6668 using gravity flow columns (Pierce) and incubated for 1 h at 4°C on a rotator. The flow through was collected and applied on a second affinity column casted from 500 μl bead suspension with immobilized VI16832 and further incubated for 1 h. Columns were washed with 20 column volumes (CV) cell lysis buffer followed by a wash with 20 CV cell lysis buffer with 150 mM NaCl instead of initial 1 M NaCl and a final wash with 20 CV 50 mM HEPES pH 7.5. Bound proteins were eluted with 20 CV prewarmed 0.5% (m/v) SDS and 5 mM DTT in 1 ml fractions. Collected fractions were pooled and lyophilized. Experiments were carried out in three independent biological replicates.

### Protein digest and phosphopeptide enrichment

Lyophilized material was dissolved in water, immediately followed by addition of 4 volumes of pre-chilled acetone and incubation at −20°C overnight. Precipitated proteins were pelleted by centrifugation (8700 × g, 30 min), dissolved in denaturing buffer (8 M urea, 20 mM HEPES, pH 8.0), alkylated by addition of iodoacetamide to a final concentration of 5 mM for 20 min at room temperature. Samples were separated into 10 gel slices by one-dimensional SDS-PAGE using pre-casted tris-glycine 4–15% gradient gels (Biorad). Tryptic peptides were obtained by in-gel digestion followed by peptide extraction. Of the resulting peptide solutions, 10% were vacuum dried and stored at −80°C until mass spectrometry analysis. The residual 90% were vacuum-dried and peptides were dissolved in 1 ml TiO_2_-binding buffer (73% (v/v) acetonitrile, 10% (v/v) lactic acid, 2% (v/v) TFA) for phosphopeptide enrichment. 50 μl from a TiO_2_-stock solution (30 mg/ml Titansphere TiO_2_ bulk material (GL sciences) in 100% acetonitrile) were added and incubated for 20 min at room temperature. After centrifugation (1000 x g, 3 min), TiO_2_-beads were washed 4 times with 80% (v/v) acetonitrile, 2% (v/v) TFA. Phosphopeptides were sequentially eluted with 5% (v/v) NH_4_OH and 30% (v/v) acetonitrile, vacuum-dried, dissolved in 0.1% (v/v) TFA and purified with C18 StageTips (Thermo Scientific). The eluated phosphopetides were again vacuum-dried and stored at −80°C.

### Mass spectrometry analysis

LC-MS/MS analyses were carried out by an EASY-nLCII HPLC system directly coupled to a LTQ Orbitrap Velos Pro hybrid mass spectrometer (Thermo Scientific) via a nano-electrospray ion source. All lyophilized (phospho)peptide samples were dissolved in 5% (v/v) acetonitrile / 0.1% (v/v) acetic acid and applied to a 20 cm-long in-house packed C18 (Aeris Peptide 3.6 μm, pore size 100 Å; Phenomenex) analytical column. Elution of peptides was carried out with binary linear gradient from 1% (v/v) acetonitrile/0.1% (v/v) acetic acid to 75% (v/v) acetonitrile/0.1% (v/v) over a period of 46 min at a flow rate of 300 nl/min. MS was operated in data-dependent mode, each full MS scan mode (300 m/z–1700 m/z; resolution 30,000). Ions with charge states of one or unassigned charge state were excluded for MS/MS scans. Fragmentation was performed either in the linear ion trap using collision induced dissociation (CID) for the most 20 intense ions with an AGC target value of 5 × 10^3^ ions and a normalized collision energy of 35% or in the Orbitrap mass analyzer of the most 10 intense ions using higher-energy collisional dissociation (HCD) with a target value of 5 × 10^4^ ions and 40% normalized collision energy. Precursor ions selected for MS/MS analysis were dynamically excluded for repeated fragmentation for a period of 20 s.

### Processing of data obtained by mass spectrometry

Obtained .raw files were analyzed using the MaxQuant software package (version 1.3.0.5) with the integrated Andromeda search engine (Cox et al., 2011). Files were collectively searched against the reviewed human proteome deposited in UniProtKB/Swiss-Prot containing 20,252 protein entries (UniProt release 2013_04). For peptide and protein identification and quantification, the following settings were used: The variable modifications were set to methionine oxidation, phosphorylation of serine, threonine and tyrosine, and amino-terminal acetylation. Carbamidomethylation was set as fixed modification. A maximum of two missed cleavages were allowed. For peptide identification, a mass tolerance of 0.5 Da and 20 ppm was allowed for the linear ion trap and the Orbitrap mass analyzer respectively. The false discovery rate (FDR) was set to 0.01. Protein quantification was based on razor and unique peptides. Protein kinases with two independent peptide identifications including at least one unique peptide were considered. Combined log_2_-transformed SILAC ratios were taken from the MaxQuant output files and considered as reliable when they were based on values from at least two independent replicates. In rare cases were a combined SILAC ratio was only calculated based on one replicate because of the absence of a signal in the unstimulated or stimulated cell state in the other replicates, this ratio was only considered if the heavy/light intensities of at least one additional replicate indicated the same direction of regulation. The assignment of kinases to groups was according to Manning et al. (2002).

### IPA, KEGG pathway, and GO analysis

For the upstream regulator analysis, protein kinases with corresponding fold changes were imported in the Ingenuity Pathway Analysis (IPA) tool (Qiagen) and the core pathway analysis was performed. Predicted upstream regulators with *p* < 0.05 and z-scores ≤ −2 (inactive) and ≥2 (active) were considered and used for network generation.

Kinases with at least two-fold abundance difference between monocytic and macrophage-like THP-1 cells were subjected to statistical enrichment analysis of KEGG pathways and GO terms in Molecular Function (MF) or Biological Processes (BP) with DAVID Bioinformatics Resources 6.7 (Huang da et al., 2009).

### Immunoblotting

Proteins were separated by one-dimensional gel electrophoresis using precasted 4–15% TRX gradient gels (Bio-Rad) followed by transfer on PVDF membranes (Merck-Millipore). Blocking of membranes was carried out for 1.5 h with 5% (m/v) milk in PBS supplemented with 0.05% (v/v) Tween-20. Membranes were probed with primary antibodies overnight at 4°C under gentle agitation. The following primary antibodies were used: anti-c-Abl (abcam #ab16903), anti-LIMK1 (CST #3842), anti-ERK1/2 (CST #9102), anti-MEK1/2 (CST #9122), anti-MEK3 (Santa Cruz #sc-961), anti-MEK4 (Santa Cruz #sc-837), anti-CaMK1 (abcam #ab68234), anti-MerTK (Santa Cruz #sc-365499), anti-CDK1 (abcam # ab18). Bound primary antibodies were detected using fluorophore-conjugated secondary antibodies (either IRDye680RD or IRDye800CW (LI-COR)) and fluorescence readout was performed using an Odyssey infrared imaging system (LI-COR).

### Phase contrast microscopy

Phase contrast micrographs were obtained by using an Olympus CKX41 fluorescence microscope.

### Cell adhesion assay

9 × 10^5^ undifferentiated THP-1 cells per well were seeded in 6-well plates (TPP) in RPMI 1640 (Sigma-Aldrich) medium supplemented with 10% FCS (Sigma). Cells were treated with 0.25, 1 or 5 μM (5Z)-7-Oxozeaenol (5Z, Tocris) or DMSO for 1 h. PMA to a final concentration of 100 nM was added and cells present in the cell culture supernatant or adhered to the cell culture plastic were separately counted. Cells present in the supernatant were collected by centrifugation and resuspended in RPMI 1640 medium. An aliquot was mixed with trypan blue solution (LifeTechnologies) and cell viability and cell count were determined by using and automated cell counter (Countess, LifeTechnologies). Adhered cells were detached by incubation with a 5-fold concentrated ready-made trypsin solution (Biochrom) for 5 min. Trypsinization was quenched by addition of RPMI 1640 medium following by cell harvest and counting as described for cells in the supernatant.

### Cultivation and processing of *Staphylococcus aureus* for infection-related experiments

*Staphylococcus aureus* HG001 (Herbert et al., 2010) was grown in LB to an optical density (OD_540_) of 0.5 at 37°C under agitation. 30 ml of the culture were centrifuged for 5 min at 8700 × g and the resulting cell pellet was washed twice and finally resuspended in RPMI 1640 medium yielding in a concentration of 5 × 10^7^ cells/ml.

### Gentamycin protection assay

1.8 × 10^5^ THP-1 monocytes were seeded in a 24-well plate (TPP) and differentiated as described above. *Staphylococcus aureus* HG001 was applied to THP-1 macrophage-like cells at a multiplicity of infection (MOI) of 25 in conditioned RPMI 1640 medium supplemented with phenol red and 10% FCS. After various infection durations, medium was removed, cells were washed with PBS and extracellular staphylococci were killed by addition of conditioned RPMI 1640 medium (supplemented with phenol red and 10% FCS) containing 100 μg/ml gentamicin (Sigma-Aldrich) and 20 μg/ml lysosthaphin (Sigma-Aldrich) for 10 min. Cells were washed twice with PBS and subsequently lysed by addition of 1% (v/v) Triton-X 100 (Roth) in PBS. Intracellular staphylococci were spread on LB agar plates and colony forming units (CFU) were determined following incubation for 24 h at 37°C.

### Flow cytometry

9 × 10^5^ differentiated THP-1 cells were either one-time treated with 1 μM 5Z or vehicle for 1 h followed by infection with a GFP-expressing isogenic mutant of *S. aureus* HG001 pCgfp at an MOI of 25 for 2 h. Cells were subsequently washed twice with PBS and non-ingested bacteria were eradicated by lysostaphin and gentamicin treatment for 10 min with final concentrations of 20 μg/ml and 100 μg/ml, respectively. Cells were washed with PBS and detached with trypsin. Digestion was quenched by addition of RPMI 1640 supplemented with 1% (v/v) FBS. Cells were pelleted by centrifugation, washed twice with PBS, resuspended in PBS buffer containing 1% (v/v) FBS and 3.8 mM sodium azide and finally analyzed for green fluorescence on an Attune Acoustic Focusing Cytometer (LifeTechnologies).

### Quantification of secreted chemokines triggered by heat-inactivated *S. aureus*

9 × 10^5^ differentiated THP-1 cells were seeded in 6-well plates as described above. Prior of experiments, medium was exchanged with RPMI 1640 without phenol red and supplemented with 1% FCS. Cells were pretreated with 1 μM 5Z for 1 h prior addition of heat-inactivated *S.aureus* HG001. *S. aureus* cells were grown and harvested as described above. *S.aureus* cells were heat-inactivated for 1 h at 60°C and resuspended in RPMI 1640 without phenol red and supplemented with 1% FCS and either 1 μM 5Z or an appropriate amount of DMSO for control cells. *S. aureus* was applied to THP-1 cells at a MOI of 25. Following various incubation times, cell culture supernatants were harvested, passed through a 0.2 μm nitrocellulose filter (VWR) and stored at −80°C. Quantification of a selected set of chemokines from cell culture supernatants after 6 h and 48 h following addition of heat-inactivated *S. aureus* was performed using a custom ELISA Array kit (Qiagen) according to the manufacturer's recommendations. The kinetics of secretion for IL-8, GROa, MIP-1a and MIP-1b were analyzed using ELISA kits (Qiagen) according to the instruction by the manufacturer.

## Results and discussion

### Monocyte-to-macrophage differentiation is accompanied by major restructuring of the kinome, increase of the general kinome phosphorylation status and changes in the basal activation of individual kinases

**Figure 1:**
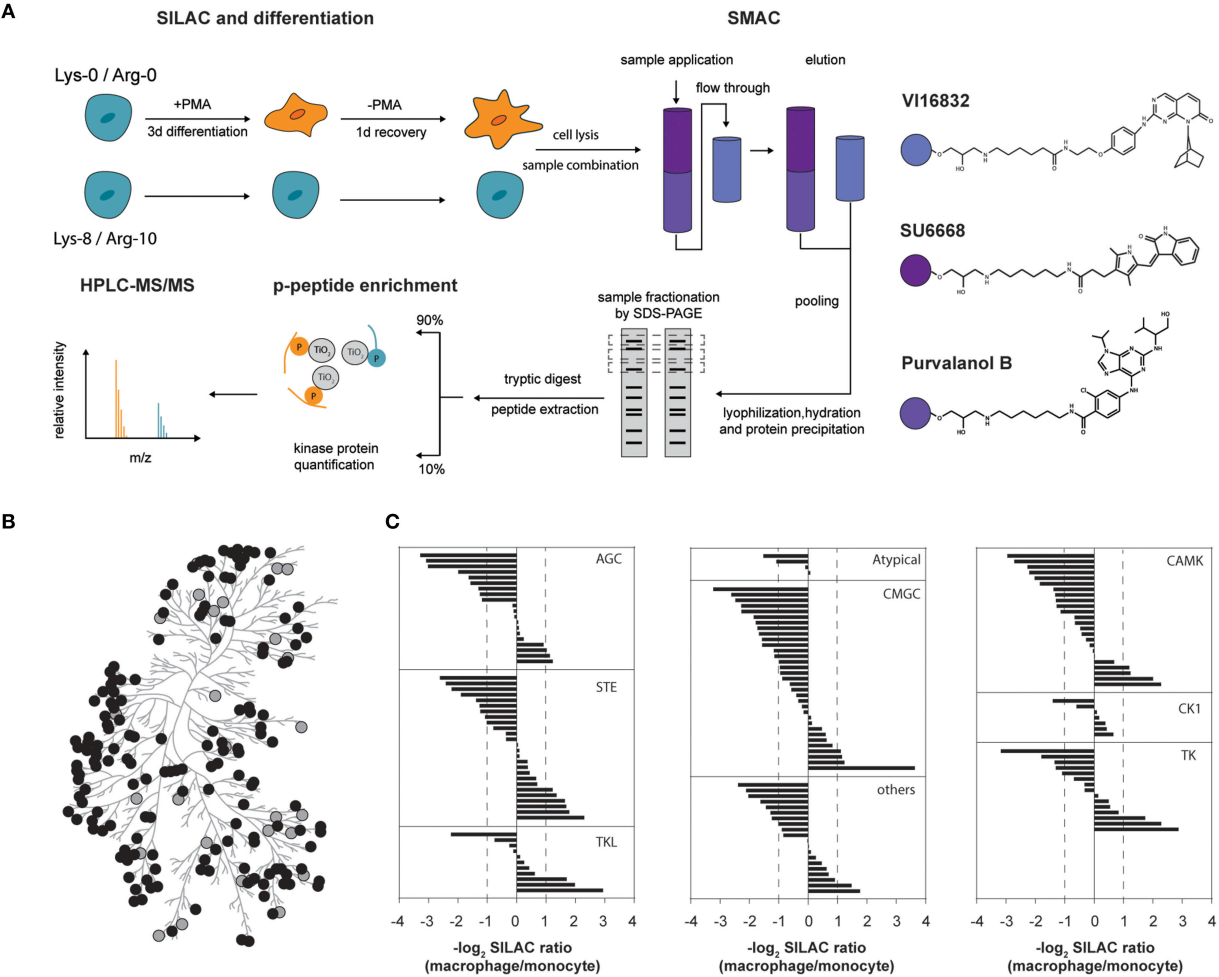
**(A)** Experimental outline for comparative kinome profiling of undifferentiated (monocytic) and differentiated (macrophage-like) THP-1 cells. Cells were metabolically labeled (SILAC) and differentiation was induced by addition of phorbol-12-myristate 13-acetate (PMA) for 3 days followed by a period of resting for 1 day in PMA-free medium. Cells were lysed and lysates from both THP-1 differentiation stages were combined equally. Small molecule affinity chromatography (SMAC) utilizing broadband kinase inhibitors SU6668, Purvalanol B and VI16832 was used for protein kinase purification. Eluted kinases were lyophilized, re-hydrated and precipitated by acetone. Gel fractionated proteins were digested by trypsin and resulting peptides were extracted. Peptides were separated whereas 90% were used for TiO_2_-based phosphopeptide enrichment prior to mass spectrometric analysis. The remainder was directly analyzed by mass spectrometry for quantification of protein kinases. **(B)** Phylogenetic kinase relationship (Manning et al., 2002). Protein kinases identified and quantified in our study are indicated as gray and black circles, respectively. **(C)** Assignment of log_2_-SILAC abundance ratios of quantified protein kinases to their corresponding kinase groups.

Cellular signaling mediated by protein kinases regulates virtually any cellular function, showing the vital role of this enzyme class for cell physiology. Despite their striking impact, protein kinases are commonly low abundant proteins and kinome characterization requires reduction of sample complexity i.e., kinase enrichment when using mass-spectrometry based proteomic strategies. Our approach to perform a comparative and system-wide kinome profiling of the human model cell line THP-1 before and after PMA-mediated induction of differentiation into the macrophage-like state is depicted in Figure 1A. We used the ATP-competitive small molecule protein kinase inhibitors Purvalanol B, SU6668 and VI16832 immobilized to sepharose beads for protein kinase family-specific pre-enrichment in combination with stable isotope labeling of amino acids in cell culture (SILAC). The inhibitors were previously demonstrated to be efficient kinase purification tools with binding of distinct but overlapping sets of protein kinases allowing a broad coverage of the kinome by their combined usage (Wissing et al., 2007; Daub et al., 2008; Oppermann et al., 2009; Zhang et al., 2013). In addition, kinase binding to mixed-inhibitor beads has recently been shown to be largely independent of kinase activity (Ruprecht et al., 2015). The enriched protein kinases were analyzed by liquid chromatography–mass spectrometry (LC-MS/MS) at the level of protein expression and site-specific protein phosphorylation, after applying an additional phosphopeptide enrichment.

Our workflow resulted in the identification of 199 protein kinases from at least two independent peptide sequences (Supplemental Table 1). Of these, 163 kinases fulfilled the criteria for a quantitative comparison between the monocytic and macrophage-like state (Figure 1B). Twenty-six protein kinases were increased at the protein level in macrophage-like cells, whereas 60 were observed with significantly higher protein amounts in the monocytic state (fold-change cut-off of 2.0). The observed changes at the level of protein affected kinases of nearly all groups of the human protein kinase-family including mitogen-activated protein kinases such as MAPK13, MAP2K1, MAP2K3, MAP2K4, MAP3K7, and MAP3K2, calcium/calmodulin-activated protein kinases e.g., CAMK1, CAMKK1, CAMK2A and CAMK2B, and Src-family kinases (FGR, HCK, SRC, YES; Figure 1C, Supplemental Table 1). The SILAC ratios of selected protein kinases with unchanged or differential expression were validated by western blots using independent protein extracts (Supplemental Figure 1).

**Figure 2:**
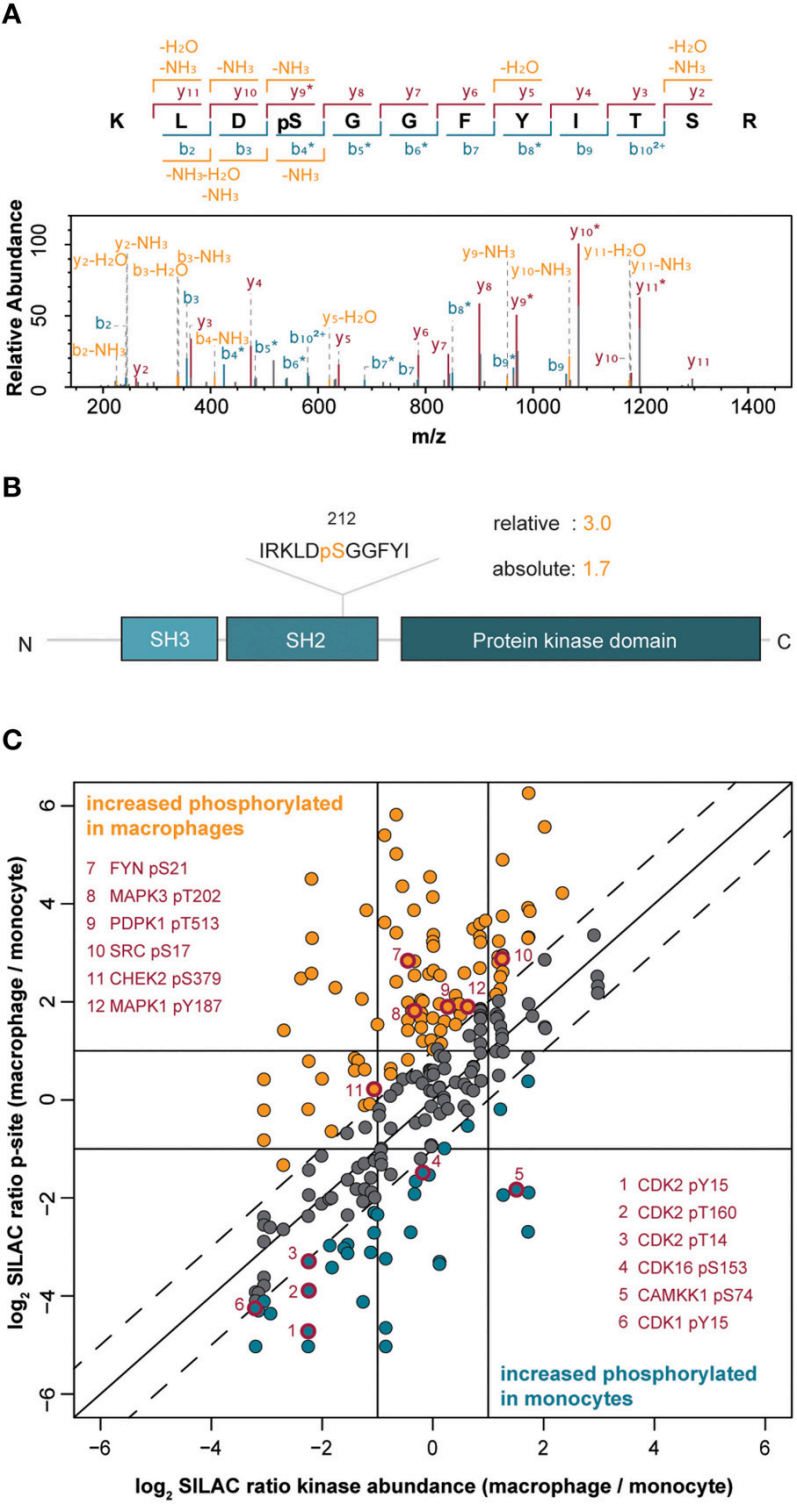
**(A)** Annotated MS/MS spectrum of the peptide from the tyrosine kinase Src spanning amino acids 209–220 with phosphorylation at Ser212. **(B)** Protein domain organization of Src indicating the pSer212 position in the SH2 domain. The difference in the level of phosphorylation of Ser212 between macrophage-like vs. monocytic THP-1 cells is indicated (relative change) and normalized to changes in the Src protein amount (absolute change). **(C)** Correlation of quantified phosphorylation sites with corresponding protein kinase abundance. The solid diagonal line indicates perfect correlation between changes in the amount of phosphorylations and changes in the corresponding protein kinase amounts, i.e., differences in phosphorylations are only attributed to changes in the protein amounts. The dashed lines indicate two-fold changes of phosphorylations (in both directions), i.e., changes in phosphorylations are not exclusively attributed to changes in protein abundance. Dots highlighted in orange indicate increased phosphorylation in macrophages whereas blue dots indicate increased phosphorylation in monocytes. Red-rimmed dots highlight kinase phosphorylation sites implicated in the induction of kinase activity.

Within the identified kinome, we mapped and quantified 311 phosphorylation sites (222 phosphoserine, 55 phosphothreonine, and 34 phosphotyrosine sites) in 118 protein kinases (Supplemental Table 1). Importantly, most sites have so far only a comparatively low number of records in which the modification was determined using site-specific methods or by proteomic discovery-mode mass spectrometry indicating that the enrichment strategy increased the sensitivity of detection of modified kinase-derived peptides with mass spectrometry (Hornbeck et al., 2012). For example, at Ser212 of the non-receptor tyrosine kinase Src, we mapped a novel phosphorylation site, located within its SH2-domain suggesting a potential regulatory role in the interaction with tyrosine phosphorylated Src interactors (Figures 2A,B). Two hundred twenty-three phosphorylation sites were considered as significantly changed following PMA-mediated differentiation when we applied the same two-fold cut-off as for the differential protein expression. Changes in phosphorylations indicated an overall increased phosphorylation status in macrophage-like cells with 142 phosphosites found increased following differentiation compared to 81 phosphosites found decreased following differentiation (Supplemental Table 1).

To assess whether the observed changes in kinase phosphorylation directly correlated with changes at the kinase expression level, we normalized the SILAC-based peptide-phosphorylation ratios of 245 phosphosites to their corresponding changes in protein expression (Figure 2C). More than half of the phosphosites still showed changes of at least two-fold after normalization proving higher or lower phosphorylation that exceeds the changes at the level of protein expression. In detail, 100 phosphosites in 52 kinases appeared as truly upregulated in the macrophage-like state and 38 phosphosites in 28 kinases were found as upregulated in monocytic THP-1 cells, further supporting a general trend toward a higher phosphorylation status following differentiation (Supplemental Table 1, Figure 2C). A comparison with the PhosphoSitePlus database (Hornbeck et al., 2012) revealed a potential functional implication for 21 of the regulated phosphosites (Supplemental Table 1). For example, in the macrophage-like cells phosphorylated Tyr687 in the receptor tyrosine kinase RET (17.3-fold up) recruits the protein-tyrosine phosphatase SHP2 and thereby contributes to activation of the PI3K/AKT pathway (Perrinjaquet et al., 2010), the monitored sites in FYN (Ser21; 9.8-fold), Src (Ser17; 2.5-fold), MAPK3 (ERK1, pT202; 4.6-fold), and MAPK1 (ERK2, pY187; 2.4-fold) are direct inducers of kinase activity (Payne et al., 1991; Schmitt and Stork, 2002; Yeo et al., 2011) suggesting an increased basal activity of these kinases, their upstream modulators as well as associated downstream pathways after cell differentiation (Figure 2C). This effect is likely independent of PMA stimulation and indeed relevant for the differentiation status, because PMA was removed and cells were rested in PMA-free media for 1 day before analysis.

In summary, our results show that monocyte to macrophage differentiation is accompanied by major rearrangements of the kinome, at the level of protein kinase expression as well as at the level of kinase phosphorylation.

### Identification of upstream regulators associated to kinome changes in monocyte differentiation

**Figure 3:**
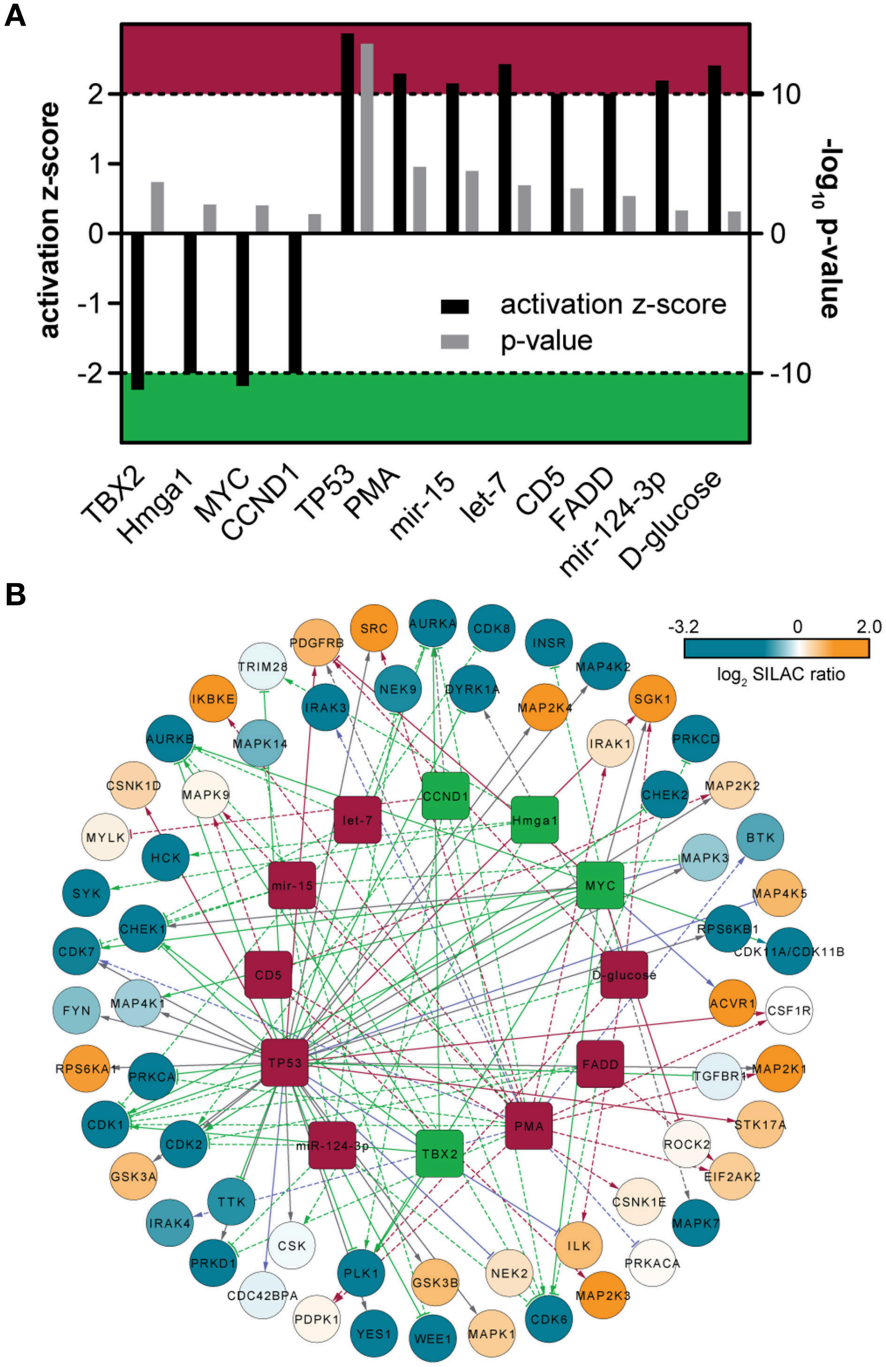
**(A)** Predicted upstream-regulators implicated in kinase expression changes. Bar chart includes activation z-score and *p*-value derived from IPA. Z-scores ≤ −2 indicate inactivity (green area), whereas z-score ≥ 2 indicate active regulators (red area). **(B)** Upstream-regulator network as predicted by IPA. The outer circle is composed of quantified protein kinases, which are color-coded according to their corresponding SILAC ratios. The inner circle is composed of predicted upstream-regulators that might be involved in the regulation of kinase expression. The upstream regulators are colored according to (A). Solid lines indicate direct molecular interaction, whereas dashed lines indicate indirect molecular interaction. Coloration of lines in orange, blue, gray, and purple indicates the predicted relationship of upstream-regulators with downstream protein kinases as activation, inactivation, no prediction and inconsistency with the state of downstream molecule, respectively.

To predict potential upstream regulators implicated in switching the monocyte/macrophage kinome at the level of gene expression, we performed an upstream regulator analysis of the quantified protein kinases with the Ingenuity Pathway Analysis (IPA) software. Twelve transcriptional upstream regulators with partly mutual regulation and overlapping downstream targets could be highlighted that are likely critical in the differentiation process (*p* ≤ 0.05, Figures 3A,B). Out of these, eight are predicted to be active (z-score ≥ 2) and four as inhibited (z-score ≤ −2). Naturally, PMA used as trigger for THP-1 cell differentiation was found among the top active regulators with a direct correlation to increased amounts of many protein kinases including MAP2K1, IRAK1, SRC, and CDK1. Among the group of transcription factors, MYC and TBX2 were predicted as inhibited with an overlap in the assigned depletion of kinases such as AURKA, AURKB, PLK1, and CDK1. Moreover, the transcriptional regulator TP53 (p53) was assigned to be active and was predicted to be involved in the transcriptional regulation of kinases including repression of DYRK1A (Zhang et al., 2011b), WEE1 (Lezina et al., 2013), PKC-alpha (Zhan et al., 2005), and induction of SGK1 (You et al., 2004). However, since expression of functional p53 has not been demonstrated for THP-1 cells so far (Sugimoto et al., 1992; Durland-Busbice and Reisman, 2002), this finding might be indicative for activation of a transcriptional regulator with a target gene pattern similar to p53. Finally, a potential significant implication for three regulatory micro-RNAs in the process of THP-1 differentiation was found, namely mir-124-3p, mir-15, and let-7, with the latter two involved in inhibition of transcripts of several cell-cycle-associated kinases including CHECK1, CDK1, CDK6, as well as AURKA, and AURKB.

Most of the suggested transcriptional regulators were not highlighted in a global transcriptome study of THP-1 cell differentiation (Consortium et al., 2009) suggesting a potential more significant relevance for kinase-coding genes that should be followed-up in future studies. However, MYC and p53, e.g., are frequently deregulated in cancer and THP-1 cells are derived from a patient with acute monocytic leukemia (Tsuchiya et al., 1980). Thus, translation of the results to primary human monocytes has to be done with care. Moreover, besides transcription, protein synthesis rates were recently determined as additional primary drivers for global protein expression changes in differentiating THP-1 cells (Kristensen et al., 2013).

### THP-1 macrophages are characterized by kinases that are related to calcium/calmodulin signaling and actin dynamics

Protein kinases increased in the macrophage state point to a prominent role of these kinases in the regulation of macrophage-specific differentiation and function and are predictors of relevant intracellular signaling pathways and downstream effects. We identified LIMK1, TNIK, MERTK, FGR, ACVR1, and STK10 within the top 15 protein kinases with highest protein ratios in macrophages vs. monocytes (12.5-fold to 3.1-fold, Supplemental Figure 1). Remarkably, the majority of these kinases collectively share functional implications in signaling pathways of actin dynamics. LIMK1 is the regulatory kinase of the actin-binding factors cofilin and destrin. The actin-depolymerizing factor (ADF)/cofilin family of proteins is essential for the dynamic changes in the actin cytoskeleton that occur during cell locomotion or other processes including phagocytosis (Arber et al., 1998; Amano et al., 2001; Bierne et al., 2001; Matsui et al., 2002). TRAF2 and NCK-interacting protein kinase TNIK regulates the c-Jun N-terminal kinase pathways, which is an essential and specific activator of Wnt target genes and regulates actin rearrangements and cell spreading (Fu et al., 1999; Taira et al., 2004; Mahmoudi et al., 2009). The tyrosine kinase Mer belongs to the unique family of TAM - Tyro3, Axl, and Mer—receptors which together with their ligands Gas6 and Protein S are essential for the efficient phagocytosis of apoptotic cells and debris and act as pleiotropic inhibitors of the innate inflammatory response to pathogens—an important prevention mechanism of chronic inflammation and autoimmunity (Scott et al., 2001; Rothlin et al., 2007). STK10/LOK has recently been demonstrated to co-localize and phosphorylate ERM (ezrin-radixin-moesin) proteins in lymphocytes, which crosslink actin filaments with plasma membranes, and is thereby important in the regulation of cell shape and migration (Belkina et al., 2009). In addition to the observed increase in protein expression, several phosphorylation sites in the aforementioned kinases showed regulation independent of the changes in protein expression (Supplemental Table 1). Whereas a single upregulated site was identified in TNIK and Fgr, STK10 was found with four up- and three downregulated sites. The upstream kinases or implication of these sites in kinase function, however, is so far unknown.

Collectively, the identified kinases indicate an increased capacity in the regulation of the actin cytoskeleton in macrophages, which is important in the control of immune responses including motility and chemotaxis, phagocytosis, and antigen presentation (May and Machesky, 2001; Van Haastert and Devreotes, 2004; Stradal et al., 2006). Severe immunodeficiency has been linked to mutations that affect cytoskeletal dynamics in macrophages and many macrophage-infecting pathogens manipulate actin remodeling to support their intracellular lifestyle (Linder et al., 2000; Krachler et al., 2011; Roy et al., 2014).

To extend our study, we evaluated the macrophage-specific kinome by performing bioinformatic enrichment analysis including gene ontology (GO) classification and Kyoto Encyclopedia of Genes and Genomes (KEGG) pathway analysis (Dennis et al., 2003; Supplemental Table 2). This approach naturally revealed the overrepresentation of kinases in individual GO-molecular functions, -biological processes and KEGG pathways. Consequently, we became aware of the significant enrichment of protein kinases (Benjamini-Hochberg corrected *p* ≤ 0.001) in terms that are associated with calmodulin-dependent protein kinase activity (GO:0004683) as well as Toll-like receptor and associated MAPK signaling (GO:0000165, hsa04620). The association of our data set to calcium/calmodulin-dependent protein kinase activity is due to the increased expression of CAMK1 (3.3-fold), CAMKK1 (2.8-fold), and CAMK2A/2B (4.9-fold/7.3-fold) when the macrophage-like state is compared to the monocytic precursor. In addition, several phosphosites were detected with changes in calmodulin-dependent protein kinases (Supplemental Table 2). Interestingly, CAMKK2, which was found with decreased expression, showed upregulated phosphorylation at Ser495 and Ser511 (4.8 and 4.0-fold) indicating increased upstream phosphorylation activity, for the latter site likely by the death-associated protein kinase (DAPK) (Schumacher et al., 2004). Components of calcium/calmodulin-dependent protein kinase cascades CAMKs operate in a variety of cellular functions including regulation of transcription activators, cell cycle, hormone production, cell differentiation, actin filament organization, and neurite outgrowth. In macrophages, CAMK1 has recently been demonstrated to be integral to the inflammatory response to sepsis (Zhang et al., 2011a) and CAMK2 has a role in antimicrobial activities via regulation of phagosome maturation (Malik et al., 2001) and is involved in the activation of the NLRP3 inflammasome, leading to cytokine production and the activation of the immune system (Okada et al., 2014). The observed overrepresentation of kinases in Toll-like receptor signaling was mainly based on MAPK cascade kinases [e.g., MAPK13 (12.5-fold), MAP3K7 (3.3-fold), MAP2K4 (2.6-fold), and MAP3K2 (2.3-fold)] reflecting their important role for the transduction of pathogen-associated signals to adequate immune responses in macrophages. The MAPK signaling-associated kinases, however, are shared by many other pathways. Interestingly, whereas the essential upstream kinases for TLR-signaling interleukin 1 receptor associated kinases IRAK 1 and 4 were below our two-fold significance cut-off, the negative regulator IRAK3 is 4.7-fold decreased in macrophage-like cells.

### THP-1 monocytes show increased expression of kinases associated with cell cycle and DNA repair

**Figure 4:**
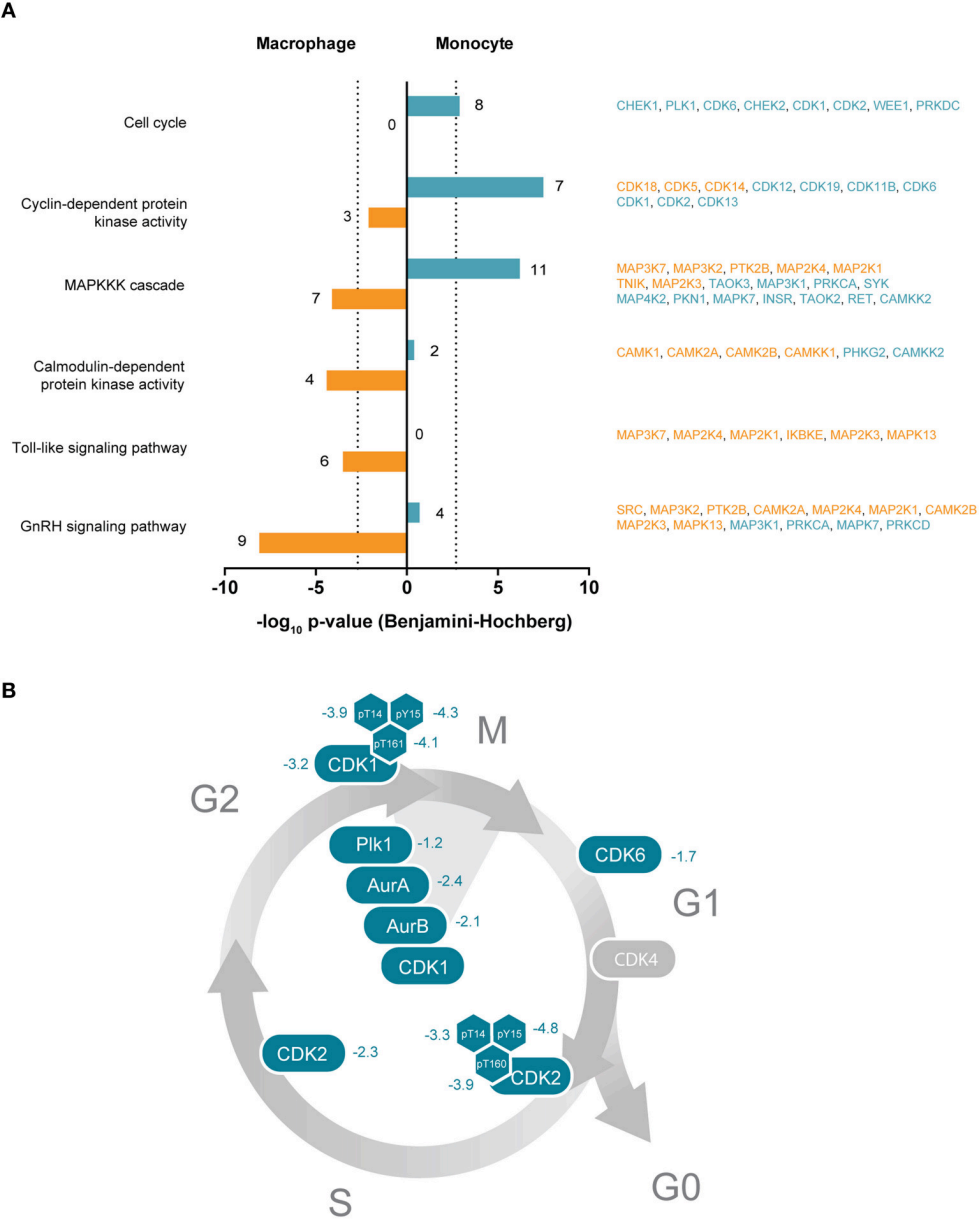
**(A)** Selected significantly enriched GO terms or KEGG pathways of protein kinases enriched either in THP-1 macrophages or monocytes. Bars indicate Benjamini-Hochberg corrected *p*-values of ≤ 0.001 (dotted line). Denoted numbers indicate the number of protein kinases assigned to the respective term or pathway. Protein kinase names are provided on the right and colored according to their affiliation to the macrophage-like (orange) or monocytic (blue) cell state. **(B)** Schematic representation of the cell cycle with important protein kinases required for cell cycle progression and mitosis. Numbers indicate determined log_2_-SILAC ratios of protein kinases and phosphorylation sites with regulatory function.

We then addressed which kinases are at higher protein levels in the monocyte state, i.e., before PMA treatment of THP-1 cells. Functional annotation analyses of the monocyte-associated kinome revealed an overrepresentation of kinases within biological processes and molecular functions related to cyclin-dependent protein kinase activity, cell cycle, MAP kinases and magnesium ion binding (Supplemental Table 2, Figure 4A). This implicates a pronounced requirement for protein kinases in the control of the corresponding signaling pathways in the monocytic cell state. In fact, the loss of proliferative activity of terminally differentiated macrophages is a hallmark of THP-1 differentiation (Auwerx, 1991). In our hands, monocyte-to-macrophage differentiation is accompanied by a pronounced decrease in the abundance of key regulatory kinases implicated in entry and progression through different cell cycle phases including the cyclin-dependent kinases CDK1, CDK2, CDK6, AURKA, AURKB, and PLK1 as well as the checkpoint kinases CHEK1 and CHEK2 (Supplemental Table 1, Figures 4A,B). In addition to the cell cycle-related kinases, several kinases involved in regulation of DNA repair mechanisms were found in significant higher amounts in the monocytic state, among them the cyclin-dependent kinase CDK9 as well as DNA-dependent protein kinase catalytic subunit PRKDC (Yu et al., 2010; Jiang et al., 2015). Several phosphosites with known functional implications, mainly in enzymatic activity, were found to be regulated in the cell cycle- and DNA repair-associated kinases [CDK2 Tyr15 and Thr160 (5.5-fold and 3.1-fold, respectively, down), CDK9 Ser464 (2.5-fold), CHEK1 Ser286 (2.2-fold) and CDK1 Tyr15 (2.1-fold)]. Phosphorylation of Thr160 in CDK2, e.g., is critical in inducing kinase activity that promotes G1/S transition and progression through S phase following binding to Cyclin E and Cyclin A respectively (Girard et al., 1991; Gu et al., 1992; Ohtsubo et al., 1995).

Our data suggest that the consequent stop of proliferation following PMA-triggered THP-1 monocyte differentiation involves depletion of cell cycle-controlling kinases in the macrophage-like cell in comparison to the monocyte precursor. The decreased relative phosphorylation level in macrophages additionally indicates downregulated upstream signaling that likely advances the proliferation arrest. Since THP-1 cells are derived from a patient with acute monocytic leukemia (Tsuchiya et al., 1980), the increased expression of cell cycle and DNA repair-associated protein kinases can be most likely linked to their oncogenic profile.

Similar to the macrophage-like characteristic kinome, we again observed an overrepresentation of kinases from MAP signaling cascades in the monocytic state, which is based on the higher protein level of a different set of members of this group including TAOK3 and MAP3K1.

### THP-1 differentiation results in rewiring of MAPK signaling networks

**Figure 5:**
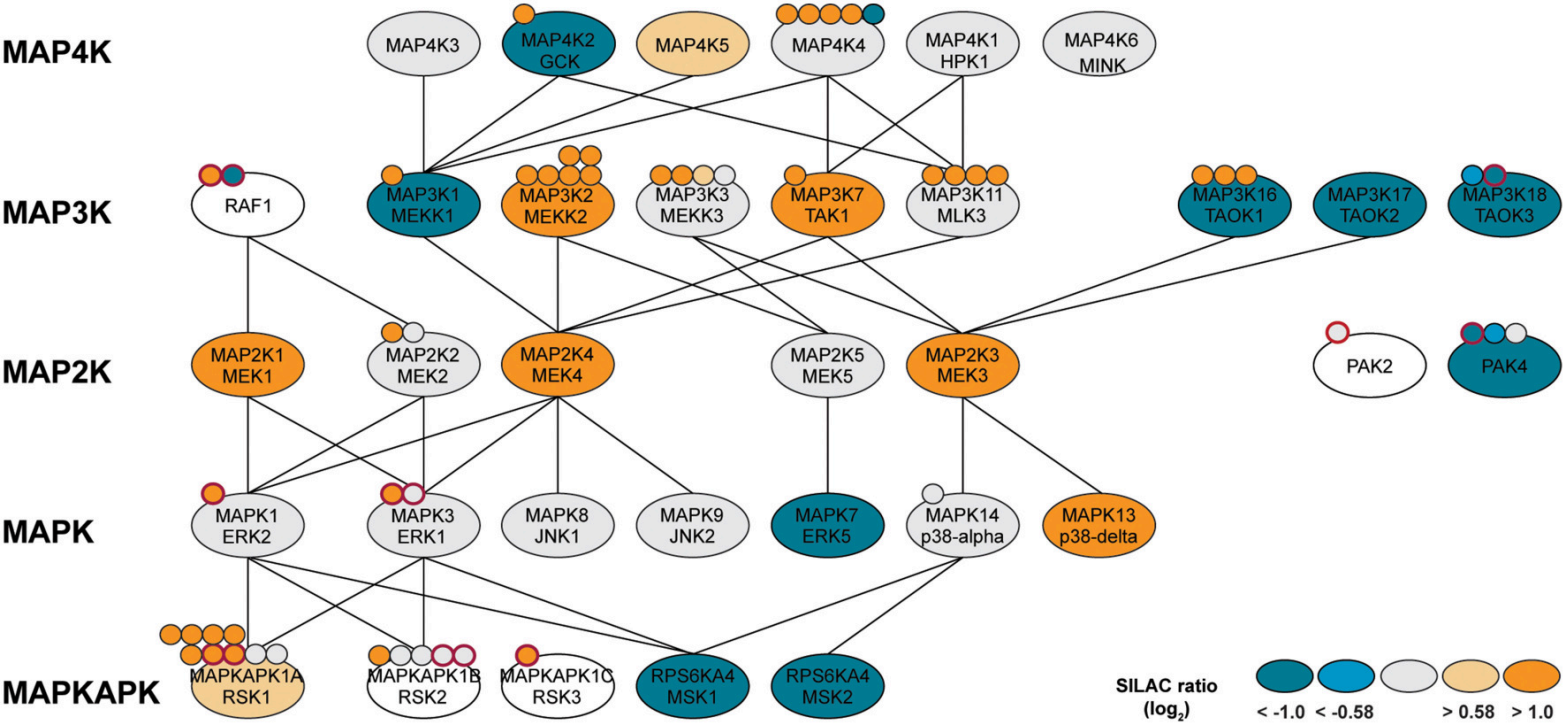
**MAP kinase network with identified protein kinases (ellipses) or phosphorylation sites (attached dots)**. Log_2_-SILAC ratios of macrophage-like and monocytic THP-1 cells are provided as color scheme. Phosphorylation sites with known functional implications are highlighted with a red border. Lines indicate kinase-substrate relationships obtained from the literature and online resources.

The noticeable overrepresentation of different sets of MAP kinases and upstream MAPK kinases prompted us to a more detailed inspection. Notably, at least 30 kinases directly associated with MAP kinase signaling cascades were identified by our approach and many showed differing amounts in THP-1 monocytes vs. macrophages (Figure 5). The diverging protein level of such a high number of MAP kinases and associates signifies the key position of this network in monocyte/macrophage differentiation and function. Differences in protein amounts of at least two-fold were observed for two MAP kinases, two MAP kinase activated protein kinases (MAPKAPKs) as well as 11 upstream MAP kinases spanning MAP4Ks, MAP3Ks, and MAP2Ks. The latter are involved in activation of MAPKs including ERK, p38alpha and JNK or NF-kappaB signaling via phosphorylation of IkappaB kinases (Matsuda et al., 1992; Yang et al., 1997; Wang et al., 2002). The MAPKs MAPK3/ERK1 and MAPK1/ERK2, MAPK8/JNK1, and MAPK9/JNK2 as well as MAPK14/p38alpha were observed at equal protein amounts and in fact only MAPK13/p38delta was found in approximately 12-fold higher amounts in macrophage-like cells, whereas ERK5 was 6.1-fold increased in monocytic THP-1 cells (Figure 5, Supplemental Table 1). However, corresponding phosphorylation profiles suggest increased basal activity for ERK1/2 (see above) but also increased activity for ERK-activated ribosomal s6 kinases (RSKs) in macrophages (Figure 5). Aside from several upstream MAPK kinases with increased expression levels (GCK, MEKK1, TAOK1/2/3, PAK4, ERK5), the mitogen- and stress-activated protein kinases MSK 1 and 2 were found in pronounced higher levels in unstimulated monocytes suggesting a more prominent function in the monocytic cell state.

Our results indicate that the differentiation of macrophage from their monocytic progenitors is accompanied by extensive rewiring of the MAPK-signaling cascades. Obviously, MAP signaling is not organized as one-way connections from one cell surface receptor via strictly separated kinase cascades to specific responsive elements but forms highly interconnected networks with overlapping hubs. For instance, p38-alpha-related signaling with downstream MSK1/2 seems more prevalent in THP-1 monocytes, whereas ERK/RSK-mediated signaling is likely more associated with the macrophage-like cell state. RSKs are multifunctional ERK effectors that regulate diverse cellular processes via cooperative regulation of many substrates including activation of the transcription factor FOS (Hauge and Frodin, 2006). MSK, on the other hand, is mainly known for its role in gene expression by phosphorylation of transcription factors such as CREB (Hauge and Frodin, 2006). In agreement with our kinase data, FOS and CREB activity is increased and decreased, respectively, following THP-1 differentiation (Consortium et al., 2009). Our data thus shed new light on how an adapted phenotype and altered signal responsiveness following differentiation can be achieved besides changing the expression of cell surface receptors or transcription factors but by fine-tuning the expression and/or activation of intermediate signaling nodes.

### MAP3K7 (TAK1) is a central signaling hub in bacterial killing, chemokine production, and differentiation activity

As a result of PMA-induced transition from the monocyte to the macrophage state, several kinases associated with the MAPK signaling network including MAP3K2 and 7 as well as MAP2K1,3 and 4, and MAPK13 were more than two-fold increased. We selected the protein kinase MAP3K7 (Transforming growth factor β-activated kinase, TAK1) for functional analysis based on its up-stream activator position of MAP2K3/4 and MAPK13 as well as the possibility to inhibit TAK1 activity by pharmacological intervention. Activation of TAK1 is triggered by various stimuli, including cytokines as well as ligands of Toll-like-, B cell- and T cell receptors, and a key signaling component of NF-κB and MAPK signaling pathways that exerts cell type-specific functions (Ninomiya-Tsuji et al., 1999; Wang et al., 2001; Wan et al., 2006; Schuman et al., 2009).

**Figure 6:**
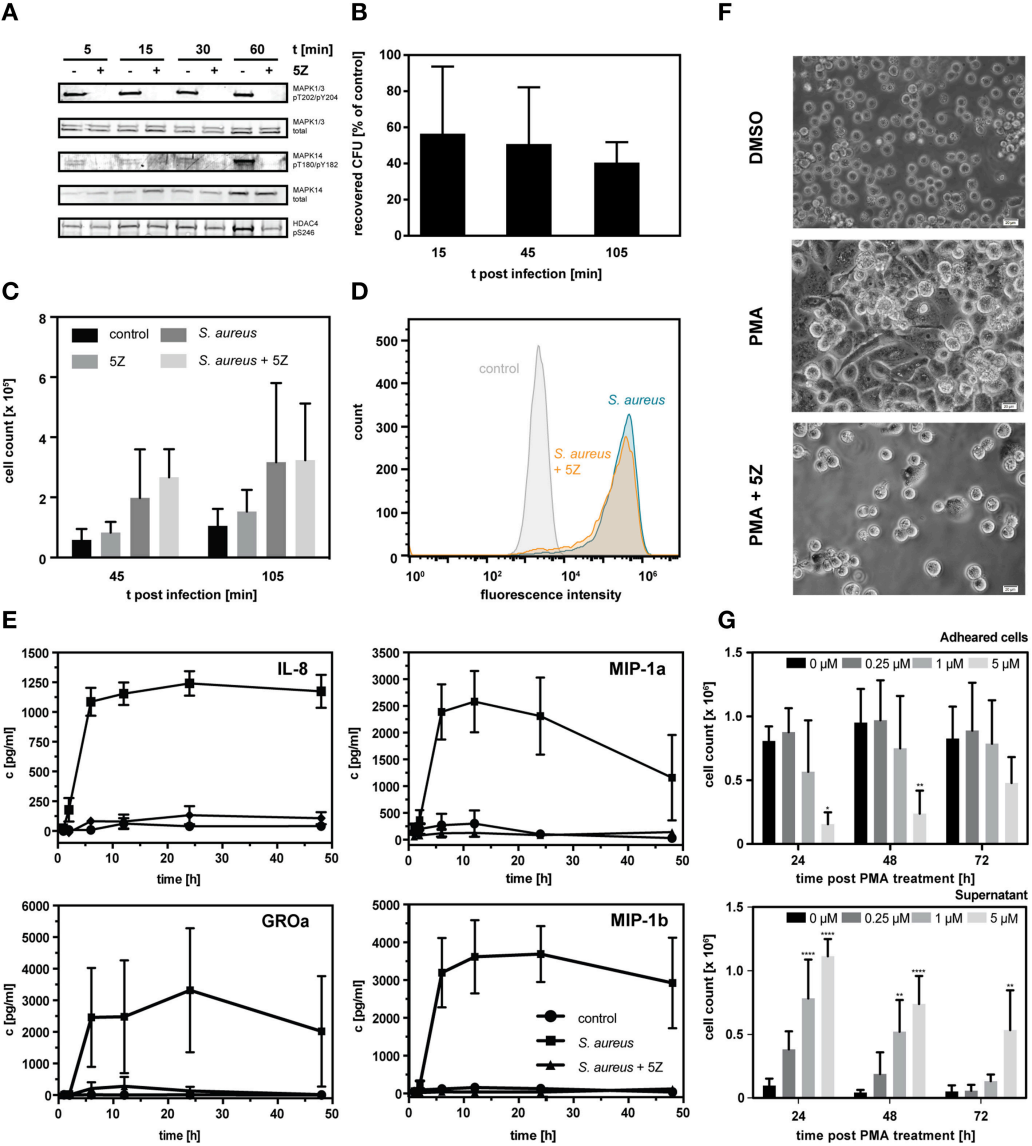
**(A)** Western blot analysis of known downstream targets of TAK1. THP-1 monocytes were treated with 1 μM 5Z or vehicle for 1 h followed by stimulation with PMA for the indicated time periods. **(B)** Percentage of recovered colony forming units (CFU) of *S. aureus* infected THP-1 cells pretreated with 1 μM 5Z normalized to untreated THP-1 cells. Data is represented as mean ± S.D. (*n* = 3). **(C)** Count of THP-1 cells present in the cell culture supernatant in uninfected controls, following infection with *S. aureus* either pretreated with 1 μM 5Z or not, and uninfected controls pretreated with 1 μM 5Z. Data is represented as mean ± S.D. (*n* = 3). **(D)** Fluorescence distribution of PMA-differentiated adherent THP-1 cells infected with GFP-expressing *S. aureus* either pretreated with 1 μM 5Z or not and uninfected control cells as analyzed by flow cytometry. **(E)** ELISA-based quantification of secreted chemokines IL-8, MIP-1a, MIP-1b, and GROa following interaction with heat- inactivated *S. aureus* either pretreated with 1 μM 5Z or not compared to control THP-1 cells. **(F)** Light micrographs of THP-1 cells 3 days post PMA stimulation either with or without pretreatment with 1 μM 5Z. **(G)** Cell counts of THP-1 cells present either in the cell culture supernatant fraction (upper chart) or adhered fraction (lower chart) monitored over 72 h post PMA treatment in the absence and presence of different concentrations of 5Z. Data is represented as mean ± S.D. (*n* = 3). Statistical analysis of variance was performed by two-way ANOVA with Bonferroni post tests. ^*^p < 0.05, ^**^p < 0.01, ^****^p < 0.0001.

From our kinomic data, we conclude TAK1 to be more active in macrophage-like cells as indicated by the increased phosphorylation of the down-stream targets MAPK1/3. Moreover, phosphorylation of MAPK1/3 (pT202/pY204) and the additional downstream targets MAPK14 (p38alpha; pT180pY182) and HDAC4 at S264 (Dequiedt et al., 2006) in short-term PMA-stimulated cells was abolished when TAK1 activity was abrogated by pre-treatment with the TAK1-selective inhibitor (5Z)-7-Oxozeaenol (5Z) (Ninomiya-Tsuji et al., 2003; Figure 6A).

To address the role of TAK1 in regulation of macrophage-associated functions, we inhibited TAK1 in differentiated THP-1 cells with a one-time treatment of 5Z and screened for effects on the efficacy in bacterial killing as well as the production of chemokines. First, we performed a gentamicin protection assay to compare the number of recovered staphylococci from infected THP-1 macrophages with and without inhibition of the TAK1 activity. Already 15 min. post infection, a prominent decrease in extracted vital *S. aureus* colony forming units derived from TAK1-inhibited THP-1 macrophages became apparent (Figure 6B). This finding of decreased surviving bacteria became significant during the course of the infection reaching a maximum of 40% 105 min post infection. Considering that recovery of *S. aureus* cells was performed only from attached THP-1 cells, we counted THP-1 cells present in the supernatant of the infection experiment to exclude that the observed strong drop in intracellular *S. aureus* is due to TAK1 inhibition-mediated increase in macrophage detachment from the cell culture surface (Figure 6C). TAK1 inhibition did not lead to significant differences in the number of cells in the suspension compared to non-TAK1-inhibited macrophages. Moreover, flow cytometric analyses with GFP-expressing *S. aureus* revealed a comparable bacterial load independent of TAK1 inhibition (Figure 6D) indicating that the efficiency of THP-1 macrophages to internalize bacteria was not altered by TAK1 activity. Collectively, the results of the infection assays thus point to a critical link of TAK1 signaling to phagocytic killing of intracellular microbes.

Next, to characterize the importance of TAK1 activity in chemokine production of macrophages, we pre-screened the secretion of an array of 12 chemokines following interaction with heat-inactivated *S. aureus* using ELISAs (Supplemental Figure 2). For seven chemokines we were able to detect a meaningful signal. While TGF-beta and MDC were secreted approximately in equal amounts in control cells and *S. aureus*-treated cells with and without 5Z, RANTES showed pronounced higher secretion in response to heat-inactivated *S. aureus* but no reduction with accompanying TAK1 inhibition. In contrast, we observed TAK1-dependent secretion of IL-8, MIP-1A, MIP-1B, and GROa which lead us to investigate kinetics of their secretion (Figure 6E). Strikingly, inhibition of TAK1 activity abolished the secretion of all four chemokines in response to *S. aureus*. The results thus indicate a pivotal role of TAK1 in the pathogen-induced production and/or release of specific chemokines following macrophage interaction with *S. aureus*.

TAK1 has recently been demonstrated to be essential for osteoclast differentiation (Lamothe et al., 2013). Eventually, we therefore asked if TAK1 has an implication in the differentiation of macrophages in the THP-1 background. For this purpose, monocytic THP-1 cells were one-time treated with 5Z prior to stimulation with PMA and monitored for indicators of differentiation including the ability to adhere to surfaces and to form characteristic morphological features. Inhibition of TAK1 activity in THP-1 monocytes by 5Z prevented typical cell elongation and formation of pseudopodia (Figure 6F), significantly reduced cellular adherence efficacy to the surface of cell culture material after PMA stimulation (Figure 6G) and caused abrogation of PMA-induced arrest in cell proliferation (Supplemental Figure 3). Cell viability, on the other hand, was not altered by TAK1 inhibition (Supplemental Figure 3).

Taken together, the obtained results highlight TAK1 as a central signaling hub involved in macrophage-associated bacterial killing and pathogen-induced chemokine production and development of a macrophage-like phenotype. Its character as central signaling component is most likely mediated through its well-known role in downstream activation of ERK, JNK, p38, and NF-kappaB signaling pathways (Tang et al., 2008; Lamothe et al., 2012; Mihaly et al., 2014). The observed prevention of PMA-induced phosphorylation of direct and indirect TAK1 downstream targets and the accompanying differentiation defect in the presence of 5Z implicates a functional cooperation of TAK1 and PKC, because PMA is an inducer of PKC activity which is essential for PMA-triggered THP-1 differentiation (Bazzi and Nelsestuen, 1989; Schwende et al., 1996). A PKC-dependent activation pathway of TAK1 has been described in lymphocytes. B cell or T cell receptor stimulation induces activation of protein kinase C-isoforms, which leads to phosphorylation of CARD11/CARMA1. A complex of CARD11/CARMA1, BCL10, and MALT1 then interacts with TRAF6 ubiquitin ligase, which in turn activates TAK1 via polyubiquitination of the TAK1 protein kinase complex composed of its binding partners, TAB1, TAB2, or TAB3 (Sato et al., 2005; Sommer et al., 2005; Schuman et al., 2009).

We also demonstrated stimulation of chemokines by the human pathogen *S. aureus* with a dependency on TAK1 activity. Our results indicate the activation of TAK1 via toll-like receptors that recognize *S. aureus*-associated molecular patterns. Different from PMA, TLRs activate TAK1 via MyD88 and recruitment of IRAK1 and IRAK4, which in turn activate the TRAF6 ubiquitin ligase leading to TAK1 activation (Shim et al., 2005). TLR2 has been shown to be the key sensor for recognition of *S. aureus* (Iwaki et al., 2002) suggesting a direct function in TAK1 activation. This, however, has not yet been experimentally demonstrated. Eventually, TAK1 inhibition let to an improved intracellular killing of *S. aureus.* This suggests subcellular alterations that increase bactericidal activity and a potential implication of reactive oxygen species (ROS) such as hydrogen peroxide. Accumulation of ROS in a non-functional TAK1 background has recently been demonstrated in different models including murine keratinocytes and intestinal epithelium as well as cells of the myeloid lineage (Omori et al., 2008, 2012; Wang et al., 2015). Increased ROS levels within phagosomes of TAK1-inhibited THP-1 macrophages could not be demonstrated thus far. From our experimental setup it cannot be excluded that TAK1 plays a similar role in stimulated monocytes. Moreover, since TAK1 is at the crossroad of multiple signaling pathways additional processes in phagosomal maturation or clearance might be affected and need to be considered in future studies addressing the specific role of TAK1-signaling in phagocytic destruction of microbes. Finally, although 5Z is a highly potent TAK1 inhibitor and selective over a set of other kinases within the MAP3K pathway, off-targets cannot be fully excluded (Kilty et al., 2013).

## Conclusion

Human monocytic THP-1 cells differentiate into macrophage-like cells with increased adherence and loss of proliferative activity by PMA treatment. We found that half of the kinome was altered at the level of protein expression and even 71% of all covered kinase phosphorylation sites were significantly changed at the level of protein phosphorylation showing a massive rearrangement of the macrophage-specific kinome in comparison to its monocytic precursor counterpart. Our analysis of the kinomic data furthermore highlights cell state-specific kinase subsets such as cyclin-dependent kinases associated with cell cycle control at higher levels in THP-1-monocytes and calmodulin-dependent kinases and kinases involved in proinflammatory signaling more expressed in macrophages. Our kinomic approach eventually revealed protein kinase MAP3K7/TAK1 as a master regulator for macrophage-associated functions. Application of functional assays allowed us to identify that TAK1 kinase activity is essentially associated with the PMA-induced arrest in cell proliferation and cellular differentiation. Moreover, we could demonstrate that TAK1 activity is associated with bacterial killing and is essential for the secretion of several chemokines including IL-8 as the primary inducer of chemotaxis in neutrophils and other granulocytes. In conclusion, we suggest the kinome rearrangement and the MAPK rewiring as a hallmark of monocyte-to-macrophage differentiation and we consider protein kinase TAK1 as a key signaling hub and master regulator for macrophage function. For future research applications, we anticipate that focus to TAK1 and its signaling network may result in the understanding of macrophage-associated diseases and may also serve as starting point for novel therapeutic targets.

## Author contributions

Conceived and designed the experiments: FH, ER. Performed the experiments: ER, MH, KV. Analyzed the data: FH, MH, ER, JM. Wrote the paper: FH, ER, JM.

## Funding

This study was fully supported by a fund from the Bundesministerium für Bildung und Forschung to FH (03Z1CN21).

### Conflict of interest statement

The authors declare that the research was conducted in the absence of any commercial or financial relationships that could be construed as a potential conflict of interest.

## Abbreviations

5Z: (5Z)-7-Oxozeaenol
CFU: colony forming units
ELISA: enzyme linked immunosorbent assay
GFP: green fluorescent protein
IPA: Ingenuity pathway analysis
KEGG: Kyoto Encyclopedia of Genes and Genomes
MAPK: mitogen activated protein kinase
MPS: mononuclear phagocyte system
PMA: phorbol-12-myristate 13-acetate
SILAC: stable isotope labeling by amino acids in cell culture
SMAC: small molecule affinity chromatography
TAK1: Transforming growth factor β-activated kinase
LC-MS/MS: Liquid chromatography–mass spectrometry.
