# Supplementary material for: Induction of Macrophage Function in Human THP-1 Cells Is Associated with Rewiring of MAPK Signaling and Activation of MAP3K7 (TAK1) Protein Kinase
Source: Front Cell Dev Biol. 2016 Mar 30;4:21. doi: 10.3389/fcell.2016.00021 (PMC4811913; doi:10.3389/fcell.2016.00021)
Supplement: Supplementary file 1 [file Supplemental_Figures.pdf]

Supplemental material

Induction of Macrophage Function in Human THP-1 Cells is Associated with Rewiring of MAPK Signaling and Activation of MAP3K7 (TAK1) Protein Kinase

Erik Richter, Katharina Ventz, Manuela Harms, Jörg Mostertz and Falko Hochgräfe*

*Correspondence: falko.hochgraefe@uni-greifswald.de

Supplemental Figure 1

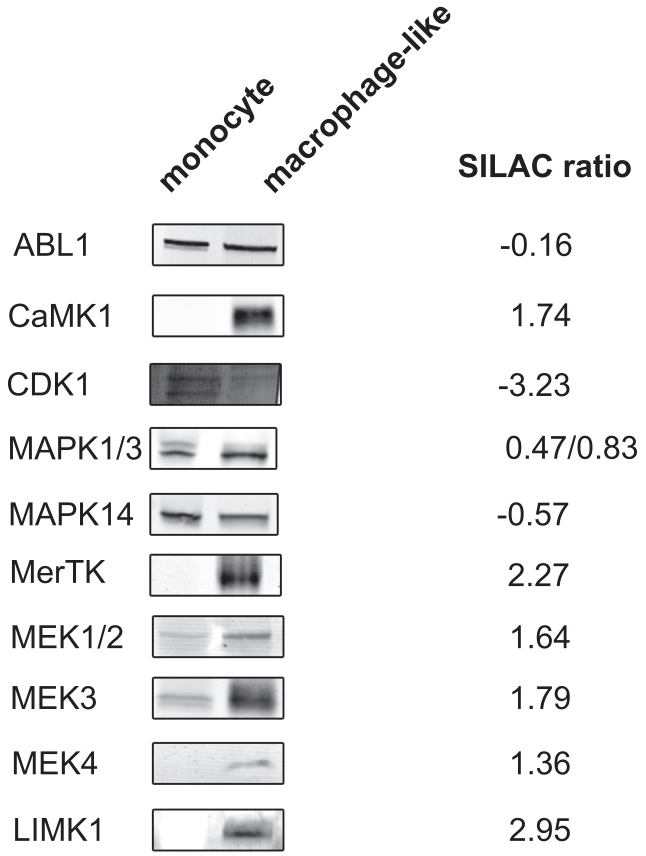

Supplemental Figure 1: Western blot analysis of selected protein kinase. Corresponding log_2-SILAC ratios (macrophage-like / monocyte) are indicated.

Supplemental Figure 2

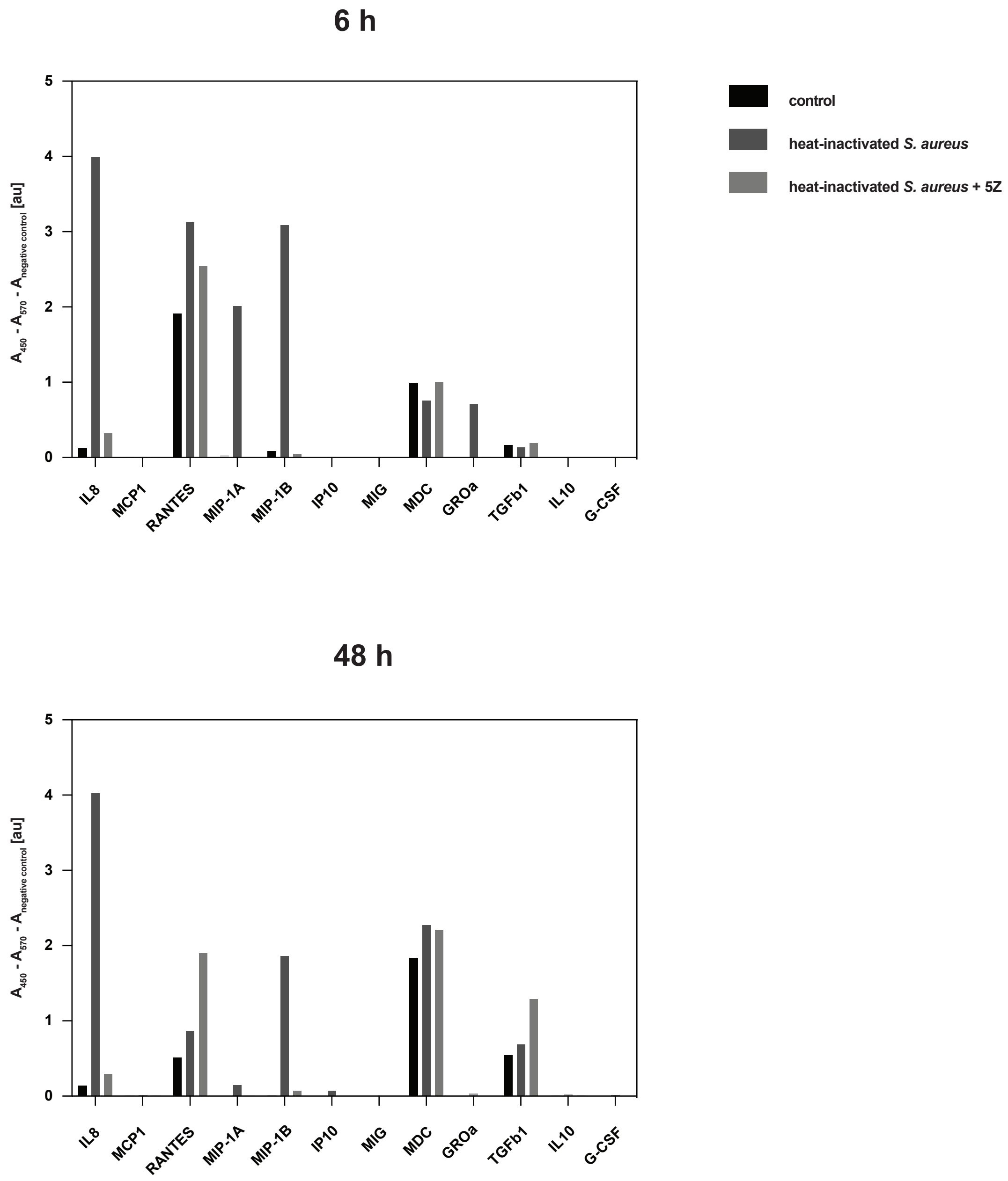

Supplemental Figure 2: Enzyme-linked immunosorbent assays of 12 chemokines in the supernatant of differentiated THP-1 cells that were either left untreated or treated with heat-inactivated *S. aureus* in the presence or absence of the TAK1-selective inhibitor 5Z.

Supplemental Figure 3

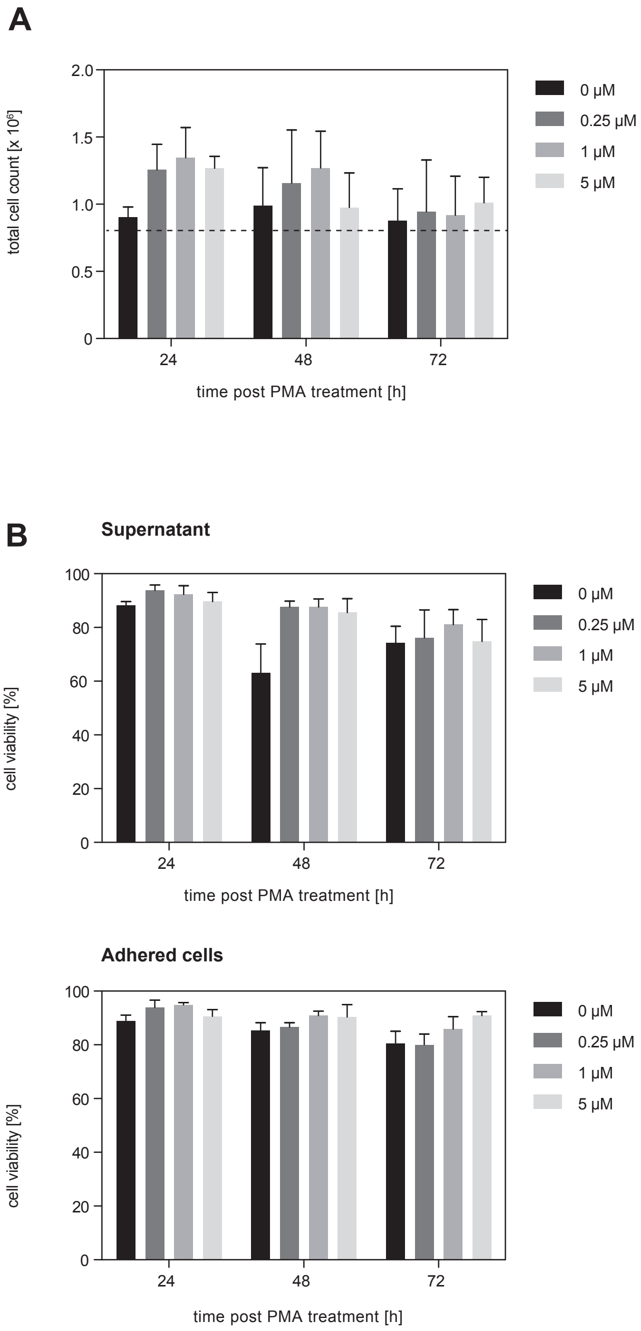

Supplemental Figure 3: Total cell counts and cell viability following treatment of THP-1 cells with PMA and 0 to 5 µM of the TAK1-selective inhibitor 5Z for 24, 48 and 72 hours. **A**: Total cell numbers, i.e. the sum of adhered cells and cells in the cell culture supernatant as represented in Figure 6G. The dashed line indicates the number of seeded cells (9 x 10^5) at t = 0 h. **B**: Cell viability of THP-1 cells in the cell culture supernatant and adherent cells corresponding to cell counts in Figure 6G.
